# Calretinin as a blood-based biomarker for mesothelioma

**DOI:** 10.1186/s12885-017-3375-5

**Published:** 2017-05-30

**Authors:** Georg Johnen, Katarzyna Gawrych, Irina Raiko, Swaantje Casjens, Beate Pesch, Daniel G. Weber, Dirk Taeger, Martin Lehnert, Jens Kollmeier, Torsten Bauer, Arthur W. Musk, Bruce W. S. Robinson, Thomas Brüning, Jenette Creaney

**Affiliations:** 1 Institute for Prevention and Occupational Medicine of the German Social Accident Insurance (IPA), Institute of the Ruhr University Bochum, Bürkle-de-la-Camp-Platz 1, 44789 Bochum, Germany; 2 Lungenklinik Heckeshorn, HELIOS Clinic Emil von Behring, Berlin, Germany; 3 Department of Respiratory Medicine, Sir Charles Gairdner Hospital, Perth, Australia; 4 School of Population Health, University of Western Australia, Perth, Australia; 5 National Centre for Asbestos Related Diseases, School of Medicine and Pharmacology, University of Western Australia, Perth, Australia

**Keywords:** Mesothelioma, Sarcomatoid, Epithelioid, Biphasic, Asbestos, Biomarker panel, Early diagnosis, Calretinin, Mesothelin, Plasma, Serum

## Abstract

**Background:**

Malignant mesothelioma (MM) is a deadly cancer mainly caused by previous exposure to asbestos. With a latency period up to 50 years the incidence of MM is still increasing, even in countries that banned asbestos. Secondary prevention has been established to provide persons at risk regular health examinations. An earlier detection with tumor markers might improve therapeutic options. Previously, we have developed a new blood-based assay for the protein marker calretinin. Aim of this study was the verification of the assay in an independent study population and comparison with the established marker mesothelin.

**Methods:**

For a case-control study in men, a total of 163 cases of pleural MM and 163 controls were available from Australia, another 36 cases and 72 controls were recruited in Germany. All controls had asbestosis and/or plaques. Calretinin and mesothelin were determined by ELISA (enzyme-linked immunosorbent assay) in serum or plasma collected prior to therapy. We estimated the performance of both markers and tested factors potentially influencing marker concentrations like age, sample storage time, and MM subtype.

**Results:**

Calretinin was able to detect all major subtypes except for sarcomatoid MM. Calretinin showed a similar performance in Australian and German men. At a pre-defined specificity of 95% the sensitivity of calretinin reached 71% and that of mesothelin 69%, when excluding sarcomatoid MM. At 97% specificity, the combination with calretinin increased the sensitivity of mesothelin from 66% to 75%. Sample storage time did not influence the results. In controls the concentrations of calretinin increased 1.87-fold (95% CI 1.10–3.20) per 10 years of age and slightly more for mesothelin (2.28, 95% CI 1.30–4.00).

**Conclusions:**

Calretinin could be verified as a blood-based marker for MM. The assay is robust and shows a performance that is comparable to that of mesothelin. Retrospective analyses would not be limited by storage time. The high specificity supports a combination of calretinin with other markers. Calretinin is specific for epithelioid and biphasic MM but not the rarer sarcomatoid form. Molecular markers like calretinin and mesothelin are promising tools to improve and supplement the diagnosis of MM and warrant further validation in a prospective study.

**Electronic supplementary material:**

The online version of this article (doi:10.1186/s12885-017-3375-5) contains supplementary material, which is available to authorized users.

## Background

Malignant mesothelioma (MM) is a highly aggressive tumor of the serous membranes with an unfavorable prognosis. Clinical symptoms are often nonspecific and in most cases the tumor is detected at an advanced stage. Early detection, preferable with noninvasive or minimally-invasive methods, could improve therapeutic approaches and outcomes.

MM is typically associated with a previous exposure to asbestos; with a latency period of 20 to 50 years. Asbestos has been classified as a human carcinogen by the International Agency for Research on Cancer (IARC) for nearly 30 years and subsequently, its production, processing, and use has been restricted or banned in many countries [1, 2]. However, a global asbestos ban as a measure of primary prevention does not yet exist and a number of nations still produce and/or use asbestos on a large scale. This continued use, coupled with the long latency between exposure and tumor incidence means that the number of new MM cases is still increasing. In Germany it is expected that the peak incidence of MM cases will occur around 2020 [3], and a similar trend is predicted for Australia [4].

In both Australia and Germany medical surveillance is offered to occupationally asbestos-exposed people for early detection of cancer (secondary prevention) and those that develop MM receive compensation. A surveillance program aimed at an early detection of MM could improve therapy options if, as in some other cancers, early diagnosis and therapy improves survival. Currently, diagnosis of MM requires tissue or cellular material that is examined by a specially trained pathologist, usually applying a panel of immunohistochemical markers [5]. Therefore, minimally-invasive procedures such as simple blood tests could greatly improve prognosis if early detection and treatment become possible [6].

A number of blood-based biomarkers for the detection of MM has been described, however, no single marker has sufficient sensitivity to detect all tumors, particularly the sarcomatoid subtype [7]. Thus, there is still a need for additional novel biomarkers, e.g., to offer more options for the assembly of marker panels with sufficient sensitivity and specificity [8].

Previously, we have developed an assay to detect calretinin in serum and plasma samples [9]. Calretinin is a 29 kDa calcium-binding protein originally found in neurons that is also expressed on the surface of mesothelial cells and overexpressed in MM [10–12]. Using primary cells from a mouse model Blum et al. demonstrated that mesothelial cell proliferation and migration was increased or decreased by overexpression or absence of calretinin, respectively, hinting at a possible target for a new therapeutic approach [13]. Calretinin is extensively used in antibody panels for the clinical diagnosis of MM by immunohistochemistry, including the sarcomatoid subtype [5, 11, 14, 15]. We found, in a small number of samples, that soluble calretinin was elevated in the blood of individuals with MM relative to healthy and asbestos-exposed controls [9]. A difference between plasma and serum samples was not evident and the antigen showed a high stability.

We now present data on the verification of the calretinin assay in a larger and independent study population from Australia and Germany and compare its performance with that of the established marker mesothelin [7, 16–21]. We also addressed the specific question of the utility of calretinin for identifying MM cases of sarcomatoid histology in blood, which was not answered in the previous study.

## Methods

### Study population and collection of samples

We used a case-control design to address specific questions. Firstly, to determine the performance of the calretinin assay for different MM histologies, we selected a series of male cases (*n* = 83) from Australia with similar numbers of samples with epithelioid (*n* = 27), biphasic (*n* = 28) or sarcomatoid (*n* = 28) histology. To enrich the number of cases of sarcomatoid histology it was necessary to use samples collected up to 15 years previously. A random selection of samples, stored for a similar length of time from age-matched individuals from Australia with benign asbestos-related disease (for reason of homogeneity, we selected pleural plaques only) was used as reference group (*n* = 88). These cases and controls are referred to as group 1 (Table 1).

**Table 1:** Characteristics of the study population (male cases and controls from Australia and Germany)

Characteristics	Australia				Germany	
	Group 1		Group 2		Group 3	
	Mesothelioma	Controls	Mesothelioma	Controls	Mesothelioma	Controls
Total	83	88	80	75	36	72
Histological subtype						
Epithelioid	27 (32.5%)		48 (60.0%)		28 (77.8%)	
Sarcomatoid	28 (33.7%)		0		0	
Biphasic	28 (33.7%)		12 (15.0%)		4 (11.1%)	
Not specified	0		20 (25.0%)		4 (11.1%)	
Pathologic changes in controls						
Plaques		88 (100%)		55 (73.3%)		0
Asbestosis		0		0		44 (61.1%)
Asbestosis and plaques		0		20 (26.7%)		28 (38.9%)
Year of blood drawing	2005	2006	2012	2012	2011	2010
Median (range)	(1996–2011)	(2000–2010)	(2011–2013)	(2011–2013)	(2008–2014)	(2009–2014)
Age at blood drawing [years]						
Median (range)	70 (53–84)	69.5 (52–84)	70 (41–89)	72 (53–90)	70 (34–85)	71 (43–83)
Calretinin storage time [months]						
Median (IQR)	81.5 (42.9–111)	59.8 (32.8–75.7)	17.2 (7.4–23.2)	19.3 (8.8–28.3)	3.6 (1.9–7)	10.9 (3.7–19.5)
Mesothelin storage time [months] Median (IQR)	n.a.	n.a.	21.3 (11.5–27.4)	21.5 (11.1–30.4)	7.5 (4.8–15.1)	26.8 (19.1–31.9)
Calretinin [ng/mL]						
N < limit of detection	30 (36.1%)	61 (69.3%)	20 (25.0%)	73 (97.3%)	12 (33.3%)	62 (86.1%)
Median (IQR)	0.79 (<0.28–1.70)	<0.19 (<0.09–0.63)	1.10 (<0.48–2.16)	<0.01 (<0.01- < 0.08)	1.01 (<0.33–1.74)	<0.20 (<0.08- < 0.34)
P-value^a^	0.0197		<0.0001		0.0009	
Mesothelin [nmol/L]						
N < limit of detection	0	0	1 (1.25%)	14 (18.7%)	0	0
Median (IQR)	2.65 (1.38–5.54)	0.77 (0.53–1.08)	4.06 (2.22–11.9)	1.02 (0.46–1.45)	2.01 (1.44–3.83)	1.03 (0.73–1.21)
P-value^b^	<0.0001		<0.0001		<0.0001	

Storage time, time between blood drawing and measurement of calretinin or mesothelin; *n.a*. not available, *IQR* interquartile range

^a^ *P*-values obtained from two sided Peto-Prentice test

^b^ *P*-values obtained from two sided Wilcoxon rank-sum test

Secondly, to verify our original findings that calretinin is elevated in the blood of MM patients [9] we analysed two additional independent sample sets, from Australian (group 2) and German (group 3) collections of more recent origin that represented a more typical clinical setting. Both groups were similar in composition regarding subtypes and age at blood drawing, to allow comparison of a possible influence of the country of origin as surrogate for potential differences by type of control or sample handling. To adjust for subtype composition four cases of sarcomatoid MM from Germany, which originally had 40 cases in total, were excluded. In total, group 2 consisted of 80 male MM cases and 75 matched controls, and group 3 consisted of 36 male MM cases and 72 controls. Because a large proportion of asbestos exposures, particularly heavy exposures, occurred in occupational settings, a typical but also more challenging target population of a future application of the tumor markers would consist of persons with known asbestos exposure and benign asbestos-related diseases to whom regular health examinations by social security institutions and statutory accident insurances are offered. Therefore, the controls from Germany (group 3) were selected from a surveillance cohort of the statutory accident insurances for patients with asbestosis and/or plaques. All workers had previous asbestos exposure and a recognized occupational disease based on these pathologies. In group 2 (Australia), we tried to include patients with asbestosis and plaques to have a similar control group (Table 1). Controls of all three groups were frequency matched to cases by age in 5-year groups, using the following intervals: ≤45, 46–50, 51–55, 56–60, 61–65, 66–70, 71–75, 76–80, 81–85, >85 years.

Samples from Australia were sourced from the Australasian Biospecimen Network tissue bank, which includes samples collected from patients attending the respiratory clinics of either Sir Charles Gairdner Hospital or the Hollywood Specialist Centre in Perth, Western Australia. The diagnosis of mesothelioma was established by experienced pathologists and confirmed by the Western Australian Mesothelioma Registry. The diagnosis of benign asbestos related disease (asbestosis and/or pleural plaques) was based on clinical and radiological findings. All patients were followed to confirm that the clinical pattern matched diagnosis. Blood was collected without anti-coagulant and sera stored in aliquots at −80 °C until use in assays.

German MM cases were recruited at the HELIOS Clinic Emil von Behring in Berlin. German controls with benign asbestos-related diseases were from individuals participating in the prospective validation study MoMar at 26 centers throughout Germany [22]. The final diagnosis in all patients was confirmed by experienced pathologists. Blood was collected into 9.0 ml S-Monovettes EDTA gel tubes (Sarstedt, Nümbrecht, Germany). After separation, plasma was stored at −80 °C until use.

All MM blood samples were collected prior to chemo- or radiation therapy.

### Determination of calretinin

Concentrations of calretinin in plasma and serum samples were determined as described [9]. In brief, a 1:1500 dilution of purified rabbit polyclonal anti-calretinin was used as capture antibody and a 1:5000 dilution of biotinylated polyclonal anti-calretinin as detection antibody. Samples (plasma or serum) were diluted 1:5 in Tris-buffered saline (pH 7.4) / 0.05% Tween 20, supplemented with 5 mM CaCl_2_. A volume of 100 μl of a diluted sample was used for each determination. Calretinin concentrations were determined from a standard curve of human purified recombinant calretinin (Swant, Belinzona, Switzerland) diluted between 10 and 0.08 ng/mL run in parallel on each plate. All determinations of calretinin were performed in the laboratory of the IPA.

The standard curve was obtained by four-parameter curve fitting using Softmax Pro 4.7.1 from Molecular Devices (Sunnyvale, CA, USA). The lower limit of detection (LOD) of the assay was defined as the concentration that corresponds to the following optical density (OD) at 414 nm: OD_414_ = ‘parameter A’ + 0.1 OD units. Where ‘parameter A’ (minimal value of the four-parameter curve fit function) is the background value of each microtiter plate and 0.1 OD units is the rounded 5-fold mean of the standard deviation of the zero standard.

### Determination of mesothelin

For the determination of mesothelin in serum and plasma samples, a commercially available ELISA kit (MESOMARK) by Fujirebio Diagnostics, Inc. (Malvern, PA, USA) was used according to the manufacturer’s instructions as described before [19, 23]. The assay was performed in both laboratories.

### Statistical analysis

In order to determine if soluble calretinin could be a biomarker for sarcomatoid MM we compared concentrations between about equal numbers of samples of different histological subtypes to controls with benign asbestos-related disease (pleural plaques), matched for gender (all male), age (median 70 years), and storage time (group 1 in Table 1).

To confirm the initially published results [9] we tested the assay in samples from Australia (group 2) and Germany (group 3). MM cases with sarcomatoid histology were excluded. Samples were matched for age; however, there were differences in storage time between group 2 and 3 (Table 1). All cases and controls were male, the median age was around 71 years. The Australian controls had either plaques or both, plaques and asbestosis, the German controls had either asbestosis or both, asbestosis and plaques.

Calretinin and mesothelin concentrations were presented with median and interquartile range (IQR). A relatively large number of calretinin concentrations were below the limits of detection (LOD), which affects the calculation of percentiles. Therefore, we marked a percentile estimated below LOD by a less-than (<) sign (Table 1). For the depiction of the scatterplots we set values below LOD to two-thirds of LOD (2/3*LOD).

Biomarker classification performance was determined by nonparametric and parametric estimation of the ROC curve with the area under the curve (AUC) estimated to assess a marker’s sensitivity for varying values of specificity. Because empirical ROC curves and AUCs are biased if LODs are present we used parametric ROC curves based on bi-lognormal or bi-Weibull distribution, which leads to proper (less biased) estimators [24].

The Peto-Prentice test was used to compare the distribution of calretinin measurements between groups [25, 26]. The Peto-Prentice test is a linear rank test developed for right-censored variables. Therefore, for LOD the variables were flipped into right-censored variables as described by Helsel [27]. Two-sample Wilcoxon Rank-Sum test was applied for comparison of the distribution of mesothelin values between groups. The chi-square test was performed to compare AUCs. Kendall’s tau (*r*_*K*_ *)* was calculated as nonparametric correlation measure between left-censored marker values, age, and storage time [27, 28].

Statistical analyses were performed using SAS/STAT and SAS/IML software, version 9.3 (SAS Institute Inc., Cary, NC, USA).

## Results

### Discrimination of MM subtypes – Marker concentrations in sarcomatoid MM

**Fig. 1:**
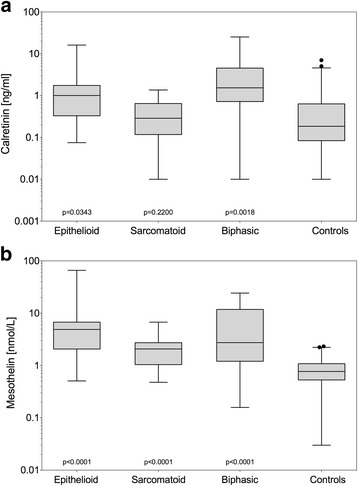
Marker concentrations in MM subtypes. **a** Calretinin [ng/mL] in controls and MM cases by subtype. **b** Mesothelin [nmol/L] in controls and MM cases by subtype. All cases and controls were from Australia (group 1). Individual *p*-values relate to the comparison between each subtype and the controls. *P*-values for calretinin were obtained from two-sided Peto-Prentice test and for mesothelin from two-sided Wilcoxon rank-sum test

The median calretinin concentration of 0.79 ng/mL in all MM cases, i.e. all subtypes combined, was significantly different (*p* = 0.0197) from the controls (<0.19 ng/mL) (group 1 in Table 1). Median calretinin concentrations for epithelioid, sarcomatoid, and biphasic MM were 1.00 ng/mL, 0.29 ng/mL, and 1.53 ng/mL respectively. The difference between controls and epithelioid (*p* = 0.0343) or biphasic MM (*p* = 0.0018) was statistically significant (Fig. 1). There was no statistical significant difference in calretinin concentrations between MM cases with sarcomatoid histology and the controls (*p* = 0.2200). Differences between sarcomatoid and epithelioid MM (*p* = 0.0041) as well as sarcomatoid and biphasic MM (*p* = 0.0001) were statistically significant.

In contrast, for mesothelin differences between controls and MM were statistically significant (*p* < 0.0001) for all individual subtypes (Fig. 1). The median mesothelin concentrations for epithelioid, sarcomatoid, and biphasic MM were 4.89 nmol/L, 2.07 nmol/L, and 2.74 nmol/L, respectively. In the controls, the median of mesothelin was 0.77 nmol/L. Differences between sarcomatoid and epithelioid MM (*p* = 0.0005) were statistically significant whereas differences between sarcomatoid and biphasic MM (*p* = 0.0963) were not.

### Verification of the performance of calretinin and influence of country of origin on the markers

To address the question whether the initial results of the calretinin assay that had been obtained with French and German samples [9] could be confirmed with an independent and larger study population, we tested the assay in samples from Australia (group 2) as well as additional German samples (group 3). For this analysis samples from cases of MM with sarcomatoid histology were excluded.

**Fig. 2:**
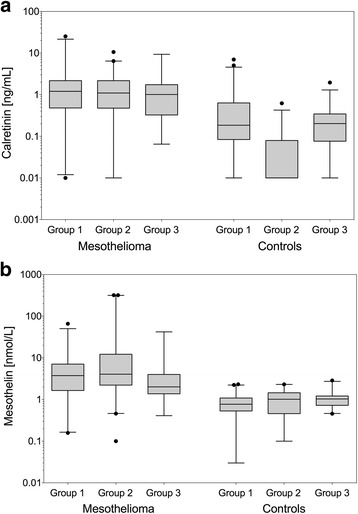
Comparison of marker concentrations in samples from Australia and Germany. **a** Calretinin [ng/mL] in MM cases and controls from Australia (group 1 and 2) and Germany (group 3). The corresponding *p*-values (group 2 vs. group 3) are: *p* = 0.8210 for MM cases and *p* = 0.0773 for controls. **b** Mesothelin [nmol/L] in MM cases and controls from Australia (group 1 and 2) and Germany (group 3). The corresponding *p*-values (group 2 vs. group 3) are: *p* = 0.0012 for MM cases and *p* = 0.1422 for controls. *P*-values for calretinin were obtained from two-sided Peto-Prentice test and for mesothelin from two-sided Wilcoxon rank-sum test. For better comparison, for group 1 sarcomatoid MM were excluded

Median calretinin concentrations in the Australian MM cases and asbestos-exposed controls were 1.10 ng/mL and <0.01 ng/mL (*p* < 0.0001), respectively (Table 1). Median calretinin concentrations in the German cases and controls were 1.01 ng/mL and 0.20 ng/mL (*p* = 0.0009), respectively (Table 1). There was no significant difference between calretinin concentrations in MM cases from Australia and Germany (*p* = 0.8210) or in controls from both countries (*p* = 0.0773) (Fig. 2a).

**Fig. 3:**
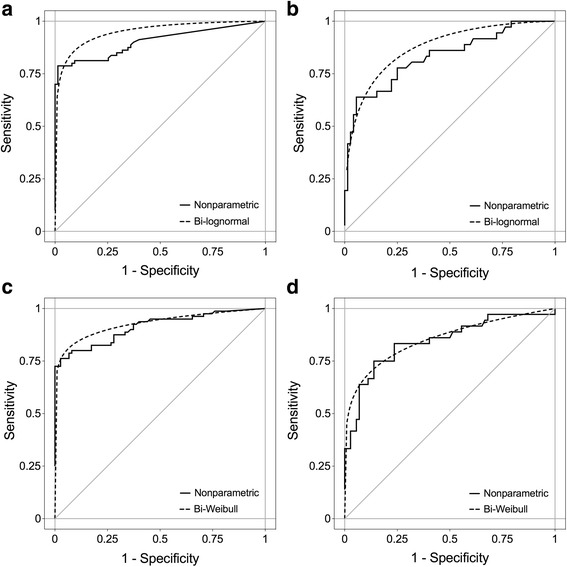
ROC analyses of calretinin and mesothelin in samples from Australia and Germany. **a** Nonparametric (AUC = 0.90, 95% CI = 0.85–0.95) and bi-lognormal (AUC = 0.95, 95% CI = 0.92–0.98) ROC curves for calretinin in Australian samples (group 2). **b** Nonparametric (AUC = 0.83, 95% CI = 0.74–0.92) and bi-lognormal (AUC = 0.87, 95% CI = 0.79–0.95) ROC curves for calretinin in German samples (group 3). **c** Nonparametric (AUC = 0.91, 95% CI = 0.87–0.96) and bi-Weibull (AUC = 0.93, 95% CI = 0.90–0.96) ROC curve for mesothelin in Australian samples (group 2). **d** Nonparametric (AUC = 0.84, 95% CI = 0.76–0.93) and bi-Weibull (AUC = 0.85, 95% CI = 0.81–0.89) ROC curve for mesothelin in German samples (group 3)

To further investigate a possible influence of the country of origin, ROC analyses were performed (Fig. 3). A relatively large number of calretinin values, particularly in the control group, were below LOD. Therefore, the ROC analyses included, besides the nonparametric (empirical), also a parametric (bi-lognormal) curve. The empirical ROC curve for Australia had an AUC of 0.90 (95% CI, 0.85–0.95) and the bi-lognormal curve an AUC of 0.95 (95% CI, 0.92–0.98). The corresponding empirical ROC curve for Germany had an AUC of 0.83 (95% CI, 0.74–0.92) and the bi-lognormal an AUC of 0.87 (95% CI, 0.79–0.95). A chi-square test with the bi-lognormal AUC indicated that between the two countries the areas were not significantly different (*p* = 0.16).

Median concentrations of mesothelin, despite being in the same order of magnitude, were different in the MM cases from Australia and Germany (*p* = 0.0012) whereas the controls were similar (*p* = 0.14) (Fig. 2b and Table 1). Again, ROC curves were used to test for a possible effect of the country where the samples originated. Figure 3 includes the nonparametric (empirical) and the parametric (bi-Weibull) ROC curves. The empirical ROC curve for Australia had an AUC of 0.91 (95% CI, 0.87–0.96) and the bi-Weibull curve an AUC of 0.93 (95% CI, 0.90–0.96). The empirical ROC curve for Germany had an AUC of 0.84 (95% CI, 0.76–0.93) and the bi-Weibull curve an AUC of 0.85 (95% CI, 0.81–0.89). A chi-square test with the AUC of the bi-Weibull curves indicated that the two areas were not significantly different (*p* = 0.17) and therefore an influence of the country of origin on the performance of mesothelin unlikely. Based on these results we pooled the data of group 2 and 3 for further analyses of the performance of the markers.

A comparison of the non-MM pathologies (plaques, asbestosis, plaques plus asbestosis) in the controls of all three groups for both markers is depicted in Additional file 1: Fig. S1. The differences between the benign asbestos-related pathologies were not statistically significant for plaques and asbestosis plus plaques as well as asbestosis and asbestosis plus plaques. The small differences between plaques and asbestosis were statistically significant for calretinin (*p* = 0.0084) as well as mesothelin (*p* = 0.0048).

### Individual and combined performance of calretinin and mesothelin to detect MM

**Fig. 4:**
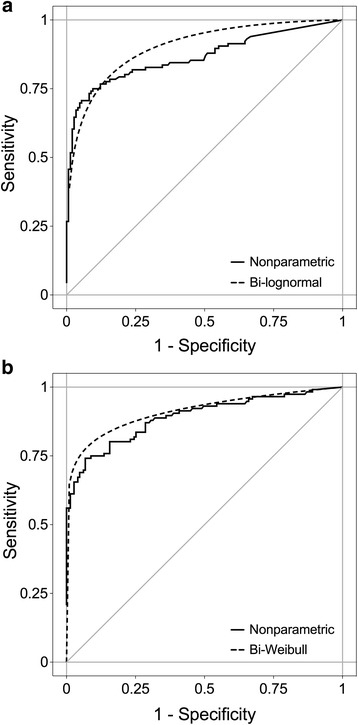
ROC analyses of calretinin and mesothelin with pooled data from Australia and Germany. **a** Nonparametric (AUC = 0.86, 95% CI = 0.82–0.91) and bi-lognormal (AUC = 0.90, 95% CI = 0.86–0.94) ROC curves for calretinin. **b** Nonparametric (AUC = 0.89, 95% CI = 0.85–0.93) and bi-Weibull (AUC = 0.91, 95% CI = 0.89–0.94) ROC curves for mesothelin. All ROC curves are based on pooled data from group 2 and 3

**Fig. 5:**
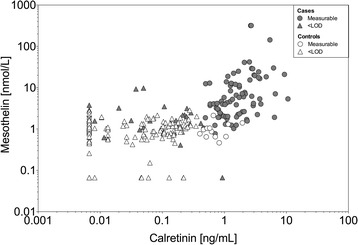
Scatterplot of calretinin versus mesothelin. The plot shows marker concentrations of MM cases and controls from Australia (group 2) and Germany (group 3)

The ROC curves (nonparametric and parametric) for calretinin and mesothelin, respectively, that were generated using the pooled dataset of male subjects of group 2 and 3 are shown in Fig. 4. Both markers indicated a good performance, with a nonparametric AUC of 0.86 (95% CI, 0.82–0.91) and a parametric AUC of 0.90 (95% CI, 0.86–0.94) for calretinin and a nonparametric AUC of 0.89 (95% CI, 0.85–0.93) and a parametric AUC of 0.91 (95% CI, 0.89–0.94) for mesothelin. Using the empirical data, specificity and sensitivity of calretinin and mesothelin were calculated for different false positive rates (FPR). Even when setting a high a priori specificity of 99% (FPR of 1%), both markers exhibit a sensitivity of over 50%, with calretinin reaching 52% and mesothelin 61% (Table 2). Accepting a FPR of 5% would lead to a sensitivity of 71% for calretinin and 69% for mesothelin. For comparison, using the maximum Youden index a sensitivity of 75% and a specificity of 90% was reached for calretinin (cutoff below LOD: 0.42 ng/mL). For mesothelin (cutoff: 1.88 nmol/L), a sensitivity of 74% and a specificity of 93% was obtained. Notably, there was a significant correlation between calretinin and mesothelin concentrations in cases (*r*_*K*_ = 0.43, *p* < 0.0001) but not in controls (*r*_*K*_ = 0.24, *p* = 0.244) (Fig. 5).

**Table 2:** Performance of calretinin and mesothelin for the detection of malignant mesothelioma in pooled data (group 2: 80 MM and 75 controls; group 3: 36 MM and 72 controls)

Biomarker	False-positive rate	Cutoff	True positive	True negative	False positive	False negative	Sensitivity	Specificity
Calretinin^a^[ng/mL]	0.01	1.07	60	145	2	56	0.52	0.99
	0.02	0.83	70	144	3	46	0.60	0.98
	0.03	0.68	78	142	5	38	0.67	0.97
	0.04	0.63	79	141	6	37	0.68	0.96
	0.05	0.58	82	139	8	34	0.71	0.95
Mesothelin^b^[nmol/L]	0.01	2.32	71	145	2	45	0.61	0.99
	0.02	2.31	71	144	3	45	0.61	0.98
	0.03	2.15	76	142	5	40	0.66	0.97
	0.04	2.02	78	141	6	38	0.67	0.96
	0.05	1.99	80	139	8	36	0.69	0.95

^a^Performance measures based on nonparametric ROC curve in Fig. 4a (AUC = 0.86, 95% CI = 0.82–0.91)

^b^Performance measures based on nonparametric ROC curve in Fig. 4b (AUC = 0.89, 95% CI = 0.85–0.93)

To assess the benefit of calretinin as an additional marker, we calculated the sensitivity gained from the combination of calretinin and mesothelin. For example, if positivity of either mesothelin (cutoff: 2.32 nmol/L) or calretinin (cutoff: 0.85 ng/mL) is sufficient for a positive test result and specificity is set to 97%, the combination reaches a sensitivity of 75% (mesothelin alone: 66%). If positivity of mesothelin and calretinin is required for a positive test result and specificity is set to 99%, the combination reaches a sensitivity of 66% (mesothelin alone: 61%).

### Influence of storage time on marker concentrations

**Fig. 6:**
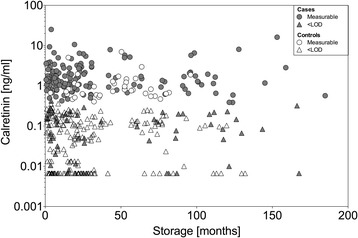
Scatterplot of calretinin versus storage time. Marker concentrations [ng/mL] in MM cases and controls from Australia and Germany (group 1, 2, and 3) were plotted against storage time [months]

For the enrichment of the rare sarcomatoid subtype in group 1 we had to resort to archival samples that were up to 15 years old at the time of marker determination. To evaluate the possible influence of storage time we looked at the distribution of assay results for calretinin in the pooled data sets of all samples (groups 1, 2, and 3), of which the latter two groups contained more of the newer samples. We observed no influence of storage time on the concentrations of calretinin (Fig. 6). There was no significant correlation between storage time and marker concentration in cases and a weak correlation in controls (cases: *r*_*K*_ = −0.05, *p* = 0.356; controls: *r*_*K*_ = 0.20, *p* < 0.0001). The odds ratio (OR) of a false-positive test for calretinin in controls was 1.02 (95% CI, 1.01–1.03). For mesothelin, storage information was only available for group 2 and group 3. Storage time did not affect mesothelin, with an OR of a false-positive test of 0.96 (95% CI, 0.92–1.01) in the pooled control group.

### Influence of patient age on the marker concentrations

**Fig. 7:**
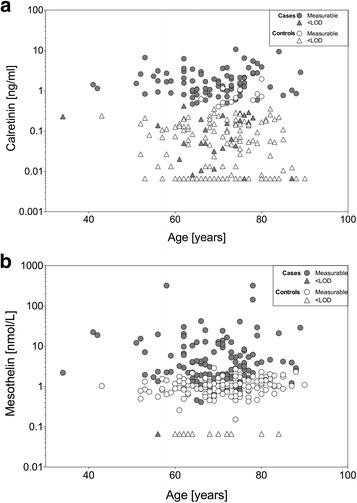
Scatterplot of marker concentrations versus age. **a** Concentrations of calretinin [ng/mL] were plotted against age [years] of MM cases and controls. **b** Concentrations of mesothelin [nmol/L] were plotted against age [years] of MM cases and controls. The plots are based on pooled data from group 2 and 3

As age can influence biomarker performance, we estimated the effect of age on the marker concentrations as shown in Fig. 7. We observed no significant correlation between calretinin and age (cases: *r*_*K*_ = 0.02, *p* = 0.782; controls: *r*_*K*_ = −0.02, *p* = 0.715) but a significant correlation of age with mesothelin in controls (*r*_*K*_ = 0.20, *p* = 0.001) but not in cases (*r*_*K*_ = 0.004, *p* = 0.954). In controls, an increase of age by ten years resulted in 1.87-fold more false-positive tests of calretinin (95% CI, 1.10–3.20) and 2.28-fold more false-positive tests for mesothelin (95% CI, 1.30–4.00).

## Discussion

Calretinin is one of the best immunohistochemical markers for the diagnosis of MM [5, 14, 15]. This prompted us to develop an assay that is independent of the availability of tissue samples and can be applied to body fluids to provide a minimally-invasive method for the detection of MM. In the current study, we have verified our initial findings [9] that calretinin is a robust blood-based biomarker significantly elevated in MM. However, the detection of sarcomatoid MM is less efficient.

### MM subtypes

Sarcomatoid MM is particularly difficult to diagnose; a blood-based biomarker elevated in MM cases of sarcomatoid histology would be clinically valuable. The Australian mesothelioma registry published that of 575 MM cases 46.8% were epithelioid, 12.2% sarcomatoid (including desmoplastic), 12.2% biphasic, and 28.3% not otherwise specified [4]. According to an analysis of the German mesothelioma registry based on more than 1600 cases, the distribution of histological subtypes in Germany consisted of 29.3% epithelioid, 9.4% sarcomatoid, and 61.3% biphasic MM [29]. Our previous study may have held some unforeseen bias as there were only 2.4% sarcomatoid cases. Results presented here, which included 28 (33.7%) sarcomatoid cases, clearly demonstrate that the calretinin assay basically does not preferentially detect sarcomatoid MM in serum. This is interesting because calretinin showed a good performance in biphasic MM and its antibody is known to detect sarcomatoid MM – including sarcomatoid areas in biphasic MM – in immunohistochemistry [11, 14, 15]. A possible explanation would be that purely sarcomatoid MM express but do not release calretinin into the bloodstream. In comparison, serum concentrations of mesothelin were somewhat, but not significantly, decreased in sarcomatoid cases and the assay did not discriminate between sarcomatoid and biphasic MM subtypes as calretinin did.

### Performance of calretinin and mesothelin to detect MM

With the exception of rare sarcomatoid cases, calretinin showed a good performance to detect MM. A slightly better performance was implicated by the parametric ROC curves, demonstrating the possible benefit of this method. A major goal of our development of markers is the future application in the screening of high-risk populations, e.g., former asbestos workers. Besides being able to detect early stages of cancer, a very high specificity is an important requirement for markers in order to avoid false-positive results that could cause unnecessary psychological burden for the participants of the screening program [30]. We therefore calculated the sensitivity of the markers for different cutoffs conditional on a specificity of at least 95%. The performance of calretinin was highly comparable to the ´gold standard´ mesothelin. When a FPR of 3% and 5% was set, calretinin showed a sensitivity of 67% and 71%, and mesothelin a sensitivity of 66% and 69%, respectively. Even when a stringent FPR of 1% was assumed, 52% and 61% of cases were detected by calretinin and mesothelin, respectively. This would render calretinin and mesothelin promising candidates for a marker panel to diagnose MM. A panel is likely to be necessary to reach sufficient sensitivity in early stages of MM. Whereas markers evaluated in case-control studies generally show higher levels because the samples are mainly derived from manifest cases that are frequently at later stages, it is expected that most marker levels will be significantly lower in patients that not yet show clinical symptoms and exhibit a small tumor mass. This has been indicated for mesothelin in a longitudinal study [19]. The loss in sensitivity is dependent upon the time between marker determination and occurrence of symptoms. A good sensitivity of markers to detect cancer within the last 12 months prior to diagnosis has been demonstrated for glycodelin and other markers in a longitudinal study based on a large trial of ovarian cancer screening [31].

### Benefit of marker combinations and other new markers

Both markers showed a good correlation. Despite of this overlap, a combination of mesothelin and calretinin improved the performance compared to mesothelin alone. Thus, calretinin appears to be a promising candidate to increase the sensitivity in a marker panel, even at high specificity. Whether other models than the simple “and” and “or” combinations we used might further improve the performance, will be the topic of a separate publication. Using logistic regression models, we have recently demonstrated that a combination of mesothelin and the microRNA miR-103a-3p in blood as well as the combination of mesothelin and hyaluronic acid in pleural effusion were able to improve the diagnostic accuracy of the assays [22, 32]. Combinations of markers from different molecular levels, e.g. proteins, methylated DNA, and microRNA as shown by Santarelli et al., appear to be a promising approach [33]. Recently, Bononi et al. discovered new circulating microRNAs that were upregulated in MM cases compared to asbestos-exposed controls; for example, miR-197-3p showed an AUC of 0.76 in the ROC analysis [34]. Another interesting candidate is the hyperacetylated isoform of the protein HMGB1, determined by mass spectrometry, reaching a maximum AUC of 1.00 when comparing serum levels of MM patients with asbestos-exposed individuals [35], while for the gene product TXN (thioredoxin) AUCs of 0.82 and 0.72 were reported by Maeda et al. and Demir et al., respectively [36, 37]. For the protein PAEP (glycodelin), originally a marker for ovarian cancer, an AUC of 0.86 was determined by Schneider et al. [38]. Regarding the detection in prediagnostic serum samples, transcript variants of the protein ENOX2 give hope that a detection of MM before onset of clinical symptoms may be feasible [39]. The new markers are promising candidates to be tested in combination with mesothelin, calretinin, or other markers. However, once verified with more cases and controls, they have to be validated in studies with longitudinal design, to finally judge their capability for early detection of MM. In addition, for some of the markers, simpler and more affordable assay formats have to be developed.

### Factors possibly influencing the marker concentration

Biomarkers have to be sufficiently robust for their application in clinical practice. Several factors may influence the concentration of markers and thus their performance [18, 40]. Factors like gender and sample matrix (serum and plasma) have been evaluated previously. We could not observe a difference by gender or matrix used in the previous study [9].

For mesothelin it has been shown that single nucleotide polymorphisms (SNPs) can affect biomarker levels [41, 42]. Because SNPs can vary between different ethnic groups – as has been shown for SNPs in metabolic enzymes by Garte et al. [43] – it cannot be excluded that markers perform differently depending on the target population. On the other hand, similar marker results from patients and controls of different geographic origin can also help to demonstrate the robustness of a biomarker. Here, we investigated regional differences by comparing samples from Australia and Germany. The comparison of calretinin concentrations in Australian and German samples from cases and controls showed no significant differences. The median concentrations of calretinin in both groups were similar and also close to the previously published values, 0.84 ng/mL for cases and 0.33 ng/mL for the asbestos-exposed controls. For comparison, the median concentration of calretinin in 97 healthy unexposed controls was 0.20 ng/mL in the previous study [9]. The results were also confirmed by the current analysis of the corresponding ROC curves, using empirical as well as parametric methods. There was some minor variation between the Australian and German controls as well as the controls of the previous analysis, which consisted solely of asbestos-exposed persons who had no benign asbestos-related diseases. The Australian controls of group 2 had either plaques (73%) or asbestosis plus plaques (27%), whereas the German controls (group 3) had mainly asbestosis (60%) or asbestosis plus plaques (39%). However, the small differences between the non-MM pathologies were statistically significant only for the comparison of plaques and asbestosis. We recruited the controls from the target population of asbestos-exposed subjects, which constitute a more challenging control group than the general population. However, a nested case-control study would be the preferred design [30, 44]. We currently conduct a prospective study in asbestos-exposed subject that may serve for the validation of calretinin, mesothelin, and other markers to detect MM.

Biobanking is an important tool for the development and evaluation of biomarkers, particularly for the validation of marker candidates in prospective cohorts. Longitudinal studies can last many years before a sufficient number of cases will be reached. A retrospective analysis of new markers might therefore be performed with archived samples and with the assumption that no significant degradation has occurred. In our study, we used serum samples that were up to 15 years old. No statistically significant influence of the storage time on the levels of calretinin could be observed. Thus, a retrospective validation of calretinin as a marker for early detection of MM within a prospective cohort study should not be limited by sample storage time. Previously, we had already demonstrated a good stability of calretinin regarding repeated freeze/thaw cycles [9]. For mesothelin it had also been shown before that storage and repeated freeze/thaw cycles did not affect the stability of the marker [23, 45].

A typical confounder of biomarkers can be the age of the target population as could be shown for the urinary marker NMP22 [46]. In the previous study on calretinin no age-related differences were observed. The current analysis revealed a moderate effect for calretinin and a slightly more pronounced effect for mesothelin with an about twofold increase of the marker concentrations by ten years of attained age. Once influencing factors have been identified and can be quantified their effect can be considered in the cutoff chosen.

### Limitations of the study

A general limitation is the case-control design of this study on the performance of biomarkers that are intended to detect MM prior to a clinical diagnosis, which tends to overestimate the sensitivity compared to a prospective design [30]. Calretinin and other markers still have to be validated in prospective cohort studies. Another limitation is the rareness of the disease so that we had to recruit archived samples. However, the biobank allowed us to include a rather large number of samples, here of male subjects.

## Conclusions

We showed that calretinin is robust and has a similar good performance to detect MM (except the sarcomatoid subtype) as mesothelin. Mesothelin is currently considered to be the best available blood-based marker for MM and therefore served as the ‘gold standard’ in our analysis. However, it is unlikely that a single biomarker will reach a sufficiently high sensitivity to allow the early detection of all MM. A panel of markers may provide the necessary increase in sensitivity, even at high specificity, as the combination of calretinin and mesothelin has indicated. This verification of calretinin provides the foundation for the next step, the validation of a specific marker panel, e.g. the combination of calretinin with mesothelin and/or other markers, in a prospective cohort study in order to prove that early detection of MM is possible. That would be a major step toward the application of biomarkers in medical surveillance programs of workers with former exposure to asbestos.

## Additional files

Additional file 1: Figure S1. Comparison of marker concentrations in different non-MM pathologies. All controls of group 1, 2, and 3 were pooled and then separated into plaques, asbestosis plus plaques, and asbestosis. *P*-values for each comparison between the three pathologies are indicated. (A) Concentrations of Calretinin [ng/mL] and (B) Mesothelin [nmol/L]. *P*-values for calretinin were obtained from two-sided Peto-Prentice test and for mesothelin from two-sided Wilcoxon rank-sum test. (TIFF 380 kb)

Additional file 2: Table S1. Dataset of the entire study population. The dataset lists histology (cases), pathologic changes (controls), age range, storage time of samples as well as measured concentrations of calretinin and mesothelin of individuals from all three study groups. (XLS 98 kb)

## Abbreviations

AUC: Area Under the Curve
CI: Confidence Interval
FPR: False Positive Rate
IQR: Interquartile Range
LOD: Limit Of Detection
MM: Malignant Mesothelioma;
OD: Optical Density
OR: Odds Ratio
ROC: Receiver Operating Characteristic
SNPs: Single Nucleotide Polymorphisms
