# Supplementary figures and images for: Calretinin as a blood-based biomarker for mesothelioma
Source: BMC Cancer. 2017 May 30;17:386. doi: 10.1186/s12885-017-3375-5 (PMC5450182; doi:10.1186/s12885-017-3375-5)

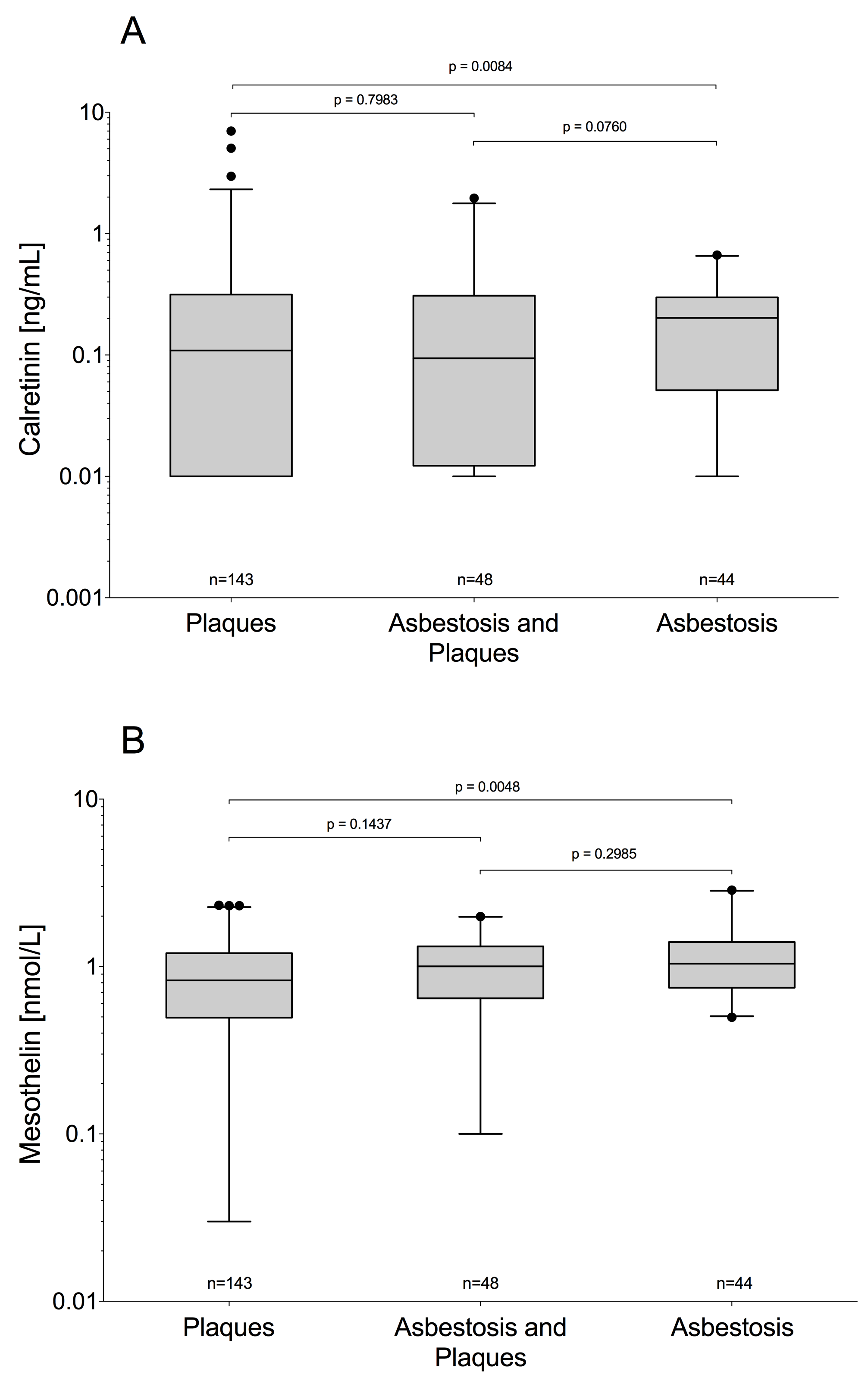

Supplement: Supplementary file 1 — Comparison of marker concentrations in different non-MM pathologies. All controls of group 1, 2, and 3 were pooled and then separated into plaques, asbestosis plus plaques, and asbestosis. P-values for each comparison between the three pathologies are indicated. (A) Concentrations of Calretinin [ng/mL] and (B) Mesothelin [nmol/L]. P-values for calretinin were obtained from two-sided Peto-Prentice test and for mesothelin from two-sided Wilcoxon rank-sum test. (TIFF 380 kb) [file 12885_2017_3375_MOESM1_ESM.tiff]
